# Account of a Locked Jaw

**Published:** 1797

**Authors:** Aaron Dexter


XXI. *Account of a Locked Jaw. By* Aaron Dexter, *M. D. F. A.A.*—From the *Memoirs of the American Academy of Arts and Sciences.* Vol. II. Part I. 4to. Boſton, 1793.

I BEG leave to preſent to the Academy a particular hiſtory of the unfortunate caſe of my friend, Dr. Edward Wyer. It rarely happens that the particular circumſtances attending the diſeaſe, with the full effect of applications, and a conſtant variation of practice, as ſymptoms appeared, can be attended to, as was the caſe in this inſtance; owing to his having no other nurſes than ſuch phyſicians, with his own aſſiſtance, as were able to change the mode according to appearances.

I preſume it may give ſome information to medical gentlemen, who have not had an opportunity of being witneſſes to ſuch diſtreſſing ſcenes.

I am, with the greateſt reſpect,

Your moſt obedient ſervant,

A. DEXTER.

The Hon. James Bowdoin, Eſq.

Preſident of the A. A. S.

*An*

*An Accurate History of a Locked Jaw, from a wounded Membrane, that terminated fatally.*

EDWARD WYER, a gentleman of the medical profession, trod upon a shingle nail, which passed through his shoe, into the ball of his left foot, directly over the tendon leading to the second toe. A few drops of blood followed: He applied only a little petroleum to the part.

The next day, (August 26, 1789) a slight lameness ensued, with nausea at the stomach. The third day, it was apparently well; and he attended his usual business without any regard to his foot.

Sunday, September 7th, whilst at dinner, he observed some difficulty in swallowing. Monday the 8th, he perceived a considerable stiffness in the muscles of his neck. On Tuesday the 9th, it increased, but not so much as to prevent his riding ten miles in the afternoon: and he concluded, he was attacked with the mumps. He applied, in the evening, volatile liniment to his neck and jaws. During the night, he was restless; and after sleeping, repeatedly found, that he had bit his tongue.

Wednesday

Wednefday morning the 10th, he perceived a ftiffnefs in his back, as though he had been fleeping on a board; and could fcarcely open his mouth. Upon his attempting to fpeak, or fwallow, fpafms feized his throat. At this time, he was fully convinced that he had a locked jaw: and the affair of the nail was recollected, with all its circumftances.

During the forenoon, his diforder increafed rapidly. At twelve o'clock, a medical friend called on him by accident; he made an effort to fpeak, but could not for fome minutes. As foon as he was able, he related his cafe, and afked advice. They converfed on the feveral different remedies that have been recommended. Opium he conceived of no avail: he faid, that he had made patients under his care, take it in large quantities. The warm bath was mentioned; but he objected to it. He agreed, that a cold bath was the only thing that could fave him. Mercurial frictions were mentioned; but he would not confent; and anfwered, that he had often tried them without any fort of advantage. He then recited the cafes in the 6th vol. of the London Medical Obfervations, proving the good effects of cold water; and, at the fame time, produced his own minutes of a cafe of a locked jaw, cured appaapparently by cold bathing, which took place about twelve months before in a neighbouring town.

On examining his foot, nothing was perceived but a very ſlight ſpeck where the nail entered. There was no ſoreneſs, tenderneſs, or pain in that part, more than in any other. It was agreed to apply the cold bath immediately. He placed himſelf on a low ſtool, naked; and two buckets of cold water were thrown on his head: after which, he placed himſelf between two blankets on his bed. An agreeable warmth ſoon took place; and he expreſſed relief, from the application, particularly at his ſtomach; and could ſwallow better.

The firſt application was about one o'clock in the afternoon; and it was repeated exactly in the ſame manner, four times.

At ſix o'clock, he was evidently relieved. After the fourth bath, he roſe from the ſtool, with great ſatisfaction; could ſpeak with eaſe; and drink without difficulty. During the five hours, he took liberally of broth and gruel. The fifth bath had a very different effect: it produced a tremor, and great anxiety. Spaſms attacked him more violently than ever, particularly on the back of his neck; which was embrocated with with oil of cloves, diluted with ſpirits of wine. The ſpaſms were very violent alſo in the muſcles of his jaws. To prevent his mouth cloſing completely, which he apprehended, he had introduced a ſtick between his teeth; and this was of great importance to him, during his life. At this time his foot was examined, and the ſpeck taken out; which did not ſhow any trace of the nail under the ſcarf ſkin. A bliſter, as ſtrong as could be made, was applied to the part: but he was utterly averſe to having the tendon examined; conceiving it too late to make any application to the part originally affected, as the diſeaſe had become general.

He wiſhed to have the ſyſtem ſupported with wine, bark, and nouriſhment.

The bath of cold water was tried again, at eight o'clock; but it evidently increaſed his diſorder; and from that time, he would never conſent to its application.

During the night, ſeveral enemas were adminiſtered, of a ſtrong decoction of red bark and ſnake root. At his uſual bed time, an anodyne of two grains of opium was given him: he paſſed a very reſtleſs night. He ſoon found, that cold drinks produced leſs ſpaſm than warm; which led him to take every thing that he was able to ſwallow, cold.

Thurſday the 11th, it was propoſed to him, to paſs a ſeton, covered with cantharides, under the ſkin of the affected part. To this he conſented, with a deſign to inflame the part: but it produced no good effects; it ſeemed rather to increaſe his irritability. In the courſe of the day, three enemas were adminiſtered of a decoction of bark, as before.

He had a great averſion to bark in any form, received into his ſtomach; as it generally, in health, produced nauſea. The object of this application was to give tone to the ſtomach; from a preſumption that debility was the immediate effect of the diſeaſe.

The gentlemen of the medical profeſſion, who were preſent, ſuggeſted to him the uſe of the warm bath; as every other application had proved of little effect: to this he conſented. About four o'clock, in the afternoon, he was placed in a bathing machine, with water heated to 90° of Fahrenheit's thermometer, in which he remained ſeventeen minutes. It produced a good effect, in relaxing the muſcles in general, particularly of his body and arms. Growing faint, he was taken out; covered with flannel; and put on his bed. A moſt profuſe diaphoreſis enſued;

ſued; and he felt ſo much relieved, that he ſaid, he then had a ſecret hope that he ſhould recover: but, within an hour, on attempting to drink ſome lemonade, his ſpaſms returned as violently as before, and were more general; but ſeemed to remit at ſhorter periods.

At eight o'clock, he was anxious to try the warm bath again; and was placed in it as ſoon as poſſible, but without any good effect. He could bear it but a few minutes, before he became faint; and ſpaſms attacked him in this ſituation. He paſſed a better night than he expected; and obtained ſome ſleep, by keeping his head accurately balanced.

Friday the 12th. This morning he ſeemed better; his ſpaſms were not ſo violent; and he was much encouraged. A laxative enema was adminiſtered; as nothing had paſſed his bowels ſince Wedneſday morning. In the afternoon, ſpaſms returned more violently than ever, and were more general. The warm bath was again uſed, but without procuring any relief: and he paſſed a very diſtreſſing night.

Saturday the 13th, a cathartic of calomel was propoſed, which met his approbation. It is to be obſerved that he now preſerved his reaſon entirely, tirely; and was unfortunately able to judge for himſelf of his ſituation, and the full effect of every application. He had, from the firſt moment, conſidered his caſe as out of the reach of medical aſſiſtance.

This morning, electricity was propoſed, which he approved; and ſuch ſparks were drawn as he was able to bear, without producing ſpaſms, from the parts moſt affected. The electric fluid was paſſed through him, in various directions, for about one hour. He thought himſelf calmer in conſequence of the application; and paſſed the day without violent ſpaſms. Electricity was repeated in the evening, but without any apparent effect. His ſenſibility had much increaſed ſince Thurſday night. Conſtant attention was neceſſary, from the phyſician, to keep the muſcles exactly balanced.

In the evening, it was agreed to make uſe of mercurial frictions; as there had been ſome ſimilar caſes related, in which this application had ſucceeded. It was uſed through the night very freely. A laxative enema was alſo adminiſtered, but without effect. His thirſt was very great. From 9 o'clock in the evening, to 6 o'clock the next morning, he drank five pints of cold water, and as much lemonade.

Sunday 14th. A discharge from his bowels was produced; but was not considered as sufficient: and ten grains of calomel were given him, with one hundred drops of laudanum. He passed a tolerable day without any violent spasms; took but little food, as his stomach nauseated it; but drank cold water and lemonade in large quantities. At 4 o'clock in the afternoon, an enema was administered of broth and half an ounce of laudanum. It was agreed to omit the mercurial frictions; and keep him as quiet as possible; and to give him as much food as could be retained on his stomach, or by his bowels. The laudanum soon had an effect. At 5 o'clock, he lay quietly sleeping under its influence. Appearances were favourable at this time, in the opinion of all the medical gentlemen present. He continued quiet, and slept easy till ten o'clock; when a laborious respiration took place. An attempt to awake him was made without effect; and the difficulty seemed to increase very fast. He was then raised up in his bed; and carried to a chair, without any signs of life, except an interrupted catching for breath, and a very feeble pulse. The most stimulating volatiles were applied to his mouth, nose, temples, &c. without any effect. At 11 o'clock, his respiration was

ſcarcely perceptible; and his pulſe intermitted. He was laid on his bed as a dead man. In a few minutes, his pulſe ſeemed more connected. He was raiſed up on the ſide of the bed; all the windows were opened; and an enema was adminiſtered, of a ſolution of cathartic ſalt in ſtrong peppermint water, which, in a few minutes, operated very largely; and part of the laudanum was evacuated. His reſpiration gradually recovered; and his pulſe roſe full. Theſe circumſtances induced his medical attendants to repeat the enema as before; at half paſt 12 o'clock, he was again placed in his bed, and breathed tolerably eaſy; and had a copious involuntary diſcharge. The external ſtimulating applications were continued; and his ſpaſms returned in a ſlight degree, juſt ſufficient to lock his jaws during their continuance. Every muſcle had been perfectly relaxed ſince 10 o'clock. His reſpiration grew gradually better; and at 3 o'clock, he was able to ſpeak; and found to his great ſurpriſe, the muſcles of his jaws relaxed, and as free from ſpaſm as ever. His thirſt was violent: he drank, from 3 o'clock to half paſt 5, one quart of cold water, two quarts of lemonade, and a bottle of ſpruce beer. After this he ſlept quietly half an hour. There ſeemed to be a fingular alteration, and he was very much elated; and fully believed, that a complete crifis had taken place. He continued free from any fpafm, particularly in his jaws and neck, till feven o'clock in the morning.

Monday 15th. At 8 o'clock his left leg and thigh were extremely affected with fpafms. The violence and pain of them were fo great, that during three hours, he was, for the firft time, deprived of his reafon. At the intervals he begged for an enema of laudanum, as the only thing that could fave him from the fevereft torture. Electricity was firft tried on the part, but without any effect. Afterwards, an enema was given with one hundred drops of laudanum. The fpafms of the abdominal mufcles forced it from him immediately. His fenfibility was fo extremely increafed, that opening a door, walking in his room, or fpeaking louder than a whifper, would produce fpafms too diftreffing for language to exprefs. Soon after the clyfter came from him, he had feveral free difcharges; and a diarrhœa took place, which lafted through the day. In the evening, a julap of oil of cinnamon, and thirty drops of laudanum, was given, which checked the difcharges; but he paffed a very reftlefs reſtleſs night. Towards morning his ſpaſms relaxed, and he ſlept a little.

Tueſday 16th. This morning he ſeemed tolerably eaſy. At 12 o'clock, a ſpaſm ſeized his diaphragm and lungs. Extreme difficulty of reſpiration came on; and he appeared to ſink under his complaints; took leave of his family; and made ſeveral arrangements reſpecting his property, and his funeral, with great compoſure: ſatisfied that, from the parts attacked, it was impoſſible for him to live but a few hours. Veſicating tincture of cantharides was applied on his breaſt; and a tea ſpoonful of Hoffman's anodyne mineral water was given him, without any effect. When life ſeemed juſt quitting him, a large diſcharge of flatus from the inteſtines, followed by a fetid diſcharge of excrement, gave immediate relief. An enema of a ſolution of cathartic ſalt was adminiſtered, which gave him two diſcharges. He ſeemed totally diſappointed in being thus relieved; and conſidered it as a ſingular medical change.

At ſeven o'clock in the evening, he took a large ſpoonful of Huxham's tincture of bark, with two ſpoonfuls of wine; which proved very grateful. His pulſe was very feeble, but his ſpaſms ſeemed to have left him altogether. It was agreed, that he ſhould repeat the laſt-mentioned medicine every hour. He aſked for cold cider, which he found very grateful to his taſte. At 8 o'clock, he repeated the tincture of bark and wine; and aſked to be turned in his bed, which was immediately done. He perceived a ſpaſm, and called for a pillow to raiſe his head a little; which being placed agreeably to his wiſh, he ſtretched himſelf out during the ſpaſm; and his reſpiration and pulſe ceaſed inſtantly, without the leaſt emotion. The medical gentlemen, who conſtantly attended him, ſuppoſed that a ſpaſm ſeized his heart, which deprived him of life.

Wedneſday morning. As it had been invariably his requeſt, that, after his death, his foot might be examined, his family conſented. The ſkin and cellular membrane were removed; and the nail could be traced to the ſheath of the tendon, which was found to have been perforated; it did not enter the tendon; but paſſed by the ſide of it to the perioſteum of the bone, which it had not affected.

Under the tendon was a ſmall cavity, about the ſize of a pea, diſcoloured throughout, with evident marks of previous inflammation.

The phalanx of the toe was taken off; but no further diſcovery was made. From Wedneſday, the 10th of September, to his death, he never was without ſome medical friend in his chamber. And from Friday morning, he had two, and frequently three or four with him. His ſituation was ſuch, that without ſome perſon well acquainted with the profeſſion, his diſtreſs muſt have been exceedingly increaſed.

